# Prognostic effect of residual plasma Epstein–Barr viral DNA after induction chemotherapy for locoregionally advanced nasopharyngeal carcinoma

**DOI:** 10.1002/cam4.6132

**Published:** 2023-05-22

**Authors:** Hua Zheng, Ping Zhou, Jun Wang, Yi‐Feng Yu, Rui Zhou, Qin Lin, San‐Gang Wu

**Affiliations:** ^1^ Department of Radiation Oncology Xiamen Cancer Center, Xiamen Key Laboratory of Radiation Oncology the First Affiliated Hospital of Xiamen University School of Medicine, Xiamen University Xiamen People's Republic of China; ^2^ Department of Head and Neck Oncology Department of Radiation Oncology, Cancer Center State Key Laboratory of Biotherapy, West China Hospital Sichuan University Chengdu People's Republic of China

**Keywords:** Epstein–Barr virus, induction chemotherapy, nasopharyngeal carcinoma, prognosis, survival

## Abstract

**Background:**

To assess the prognostic effect of plasma Epstein–Barr virus (EBV) DNA load after induction chemotherapy (post_IC_‐EBV DNA) on survival outcomes in locoregionally advanced nasopharyngeal carcinoma (LA‐NPC).

**Methods:**

Patients who were diagnosed with LA‐NPC between August 2017 and October 2021 were included. The chi‐squared test, receiver operating characteristic, Kaplan–Meier survival analysis, and Cox proportional hazard model were used for statistical analysis.

**Results:**

We included 172 patients with EBV DNA‐positive LA‐NPC in this study. There were 35.5% (*n* = 61) of patients had plasma residual EBV DNA after induction chemotherapy (IC). Patients with higher EBV DNA before IC (*p* < 0.001) and advanced nodal stage (*p* = 0.031) were significantly related to a higher rate of residual post_IC_‐EBV DNA. Patients with detectable post_IC_‐EBV DNA had inferior 3‐year locoregional relapse‐free survival (LRFS) (86.7% vs. 96.9%, *p* = 0.020), distant metastasis‐free survival (DMFS) (76.8% vs. 94.2%, *p <* 0.001), disease‐free survival (DFS) (68.2% vs. 91.1%, *p <* 0.001), and overall survival (OS) (87.8% vs. 97.9%, *p* = 0.044) compared to those with undetectable post_IC_‐EBV DNA. The multivariate prognostic analyses showed that detectable post_IC_‐EBV DNA was the independent prognostic factor related to LRFS (*p* = 0.032), DMFS (*p* = 0.010), and DFS (*p* = 0.004) than those with undetectable post_IC_‐EBV DNA. Pretreatment EBV DNA load had no prognostic effect in the multivariate analyses.

**Conclusions:**

The monitoring of plasma post_IC_‐EBV DNA has improved prognostication in LA‐NPC. Our findings suggest that post_IC_‐EBV DNA may be a robust indicator to identify the optimal candidate for intensive treatment.

## 1 INTRODUCTION

Nasopharyngeal carcinoma (NPC) is a malignant epithelial tumor with a high incidence in southern China.^1^, ^2^ Approximately 70% of patients were diagnosed with locoregionally advanced NPC (LA‐NPC) (stage III‐IVa) and induction chemotherapy (IC) plus concurrent chemoradiotherapy (CCRT) is the optimal treatment for this population based on the results from several prospective studies.^3^, ^4^, ^5^, ^6^ The tumor‐node‐metastasis (TNM) staging is used worldwide for guiding treatment selection and predicting clinical outcomes.^7^ However, the long‐term findings from the above trials indicated that approximately 20%–30% of patients would develop disease recurrence.^8^, ^9^ Thus, it is still necessary to determine robust prognostic factors that can help accurately guide the risk stratification and treatment strategy adjustment of this disease.

NPC is associated with Epstein–Barr virus (EBV) infection and plasma EBV DNA is an important indicator of EBV load in NPC.^10^, ^11^ Several studies have confirmed that plasma EBV DNA is a powerful prognostic indicator in NPC in addition to TNM staging.^12^, ^13^, ^14^, ^15^ The decrease in EBV DNA titer during treatment was found to be significantly related to the survival of a new diagnosis or metastatic NPC.^16^, ^17^, ^18^ In recent years, several studies showed that the unfavorable EBV DNA response to IC was associated with inferior survival in LA‐NPC.^19^, ^20^ Therefore, the prognostic effect of EBV DNA is brought forward to the course of IC. Here, we conduct the present study to assess the effect of plasma EBV DNA load after IC (post_IC_‐EBV DNA) on survival outcomes to provide benefits for risk‐based treatment modification in LA‐NPC.

## 2 PATIENTS AND METHODS

### 2.1 Patient selection

We retrospectively included patients who were diagnosed with NPC between August 2017 and October 2021 at our institution. Eligibility criteria included: (1) pathologically confirmed stages III–IVA NPC according to the 8th TNM staging; (2) Eastern Cooperative Oncology Group performance status of 0 or 1; (3) treated with three cycles of IC; (4) treated with intensity‐modulated radiotherapy (IMRT) and two cycles of platinum‐based CCRT; (5) plasma EBV DNA data before and after the completion of IC were available; (6) adequate haematologic, liver, and renal function. Patients with secondary malignancy, pregnancy, or lactation were excluded. Moreover, patients who did not complete radiotherapy and those who received adjuvant chemotherapy were excluded. The Ethics Committee of the First Affiliated Hospital of Xiamen University approved this study and informed consent was obtained from all patients.

### 2.2 Variables

The following patient and tumor characteristics were included in the analyses: age, gender, smoking history, histology, T stage, N stage, clinical stage, plasma EBV DNA before IC (pre_IC_‐EBV DNA), post_IC_‐EBV DNA, and IC regimens. Smoking history included never smokers and ever smokers. Ever smokers included former and current smokers, defined as patients who smoked within the last year or who had quit smoking for more than 1 year, respectively.

### 2.3 Chemotherapy and radiotherapy

All patients received three cycles of platinum‐based IC and two cycles of platinum‐based CCRT according to the institutional guideline recommendations. The IC regimens in our institution included docetaxel + cisplatin/nedaplatin (TP), docetaxel + cisplatin/nedaplatin +5‐fluorouracil (TPF), or gemcitabine + cisplatin/nedaplatin (GP). Because of the low renal toxicity of nedaplatin, we used the nedaplatin‐based IC regimen in cisplatin‐ineligible patients.^21^ Concurrent cisplatin, nedaplatin, or lobaplatin was used during CCRT based on the results from the previous prospective randomized studies.^21^, ^22^ All chemotherapy was performed every 3 weeks. All patients were treated with IMRT. The total doses to the primary nasopharyngeal tumor, metastatic neck lymph nodes, high‐risk clinical target volume, and low‐risk clinical target volume were 70 Gy/32–33 fractions (f), 66–70 Gy/32–33f, 62 Gy/32–33f, and 56 Gy/32–33f, respectively.

### 2.4 EBV DNA quantification

Peripheral whole blood samples (10 mL) were obtained before and after IC. Circulating EBV DNA was extracted from plasma and measured by droplet digital PCR (ddPCR). The results are shown as IU/mL instead of copies/mL in the present study. The threshold of post_IC_‐EBV DNA was classified as detectable (>0 IU/mL) and undetectable (0 IU/mL).

### 2.5 Follow‐up and survival outcomes

According to our institutional guideline recommendations, patients were evaluated once every 3 months in the first 3 years, every 6 months in the fourth to fifth years, and every year thereafter. Routine follow‐up examination included physical examination, plasma EBV DNA, complete head and neck, chest, abdomen, and bone‐related imaging examination. Positron emission tomography‐computed tomography was recommended for patients with detectable plasma EBV DNA or suspected of disease recurrence by imaging examination. The study endpoints included locoregional relapse‐free survival (LRFS), distant metastasis‐free survival (DMFS), disease‐free survival (DFS), and overall survival (OS). LRFS was defined as the time interval between NPC diagnosis and local or regional recurrence or both. DMFS was defined as the time interval between NPC diagnosis and distant recurrence. DFS was defined as the time interval between NPC diagnosis and disease failure or death from any cause. OS was defined as the time interval between NPC diagnosis and death from any cause.

### 2.6 Statistical analysis

Categorical variables between those with and without post_IC_‐EBV DNA were compared by the chi‐squared test or Fisher's exact test. One‐way ANOVA analysis was used to compare the continuous variable differences between groups. The area under the receiver operating characteristic (ROC) curve was performed to determine the optimal cutoff point for plasma EBV‐DNA. The difference in pretreatment EBV DNA load between those with and without post_IC_‐EBV DNA was compared by the Kruskal–Wallis test and the Mann–Whitney *U*‐test. Kaplan–Meier method was performed to calculate the survival curve, and the prognosis of patients between the subgroups was compared using the log‐rank test. Univariate and multivariable Cox regression analyses were used to determine the independent factors associated with survival outcomes. Those with a *p* < 0.10 in the univariate analysis were entered into the multivariate Cox analysis. A *p* < 0.05 was considered to be statistically significant. All analyses were conducted by the SPSS version 22.0 (SPSS Inc.), MedCalc Statistical Software version 18.2.1 (MedCalc Software bvba), or GraphPad Prism version 7.0 (GraphPad Software).

## 3 RESULTS

### 3.1 Patient baseline characteristics and survival

We included 172 patients in this study (Table 1). Of these patients, 73.8% (*n* = 127) were male, 87.2% (*n* = 150) had WHO III subtype, 72.1% (*n* = 124) had stage T3‐4 diseases, and 74.4% (*n* = 128) had stage N2‐3 diseases.

**TABLE 1:** Baseline characteristics of 172 patients with NPC.

Variables	*N*	Undetectable EBV DNA after IC (%)	Detectable EBV DNA after IC (%)	*p*
Age (mean ± SD) (years)	–	47.2 ± 11.3	46.4 ± 10.3	0.658
Gender				
Male	127	80 (72.12)	47 (77.0)	0.587
Female	45	31 (27.9)	14 (23.0)	
Smoking history				
No	88	58 (52.3)	30 (49.2)	0.751
Yes	84	53 (47.7)	31 (50.8)	
Histology				
WHO I	1	1 (0.9)	0 (0)	0.760
WHO II	21	15 (13.5)	6 (9.8)	
WHO III	150	95 (85.6)	55 (90.2)	
T stage				
T1	21	12 (10.8)	9 (14.8)	0.500
T2	27	15 (13.5)	12 (19.7)	
T3	85	56 (50.5)	29 (47.5)	
T4	39	28 (25.2)	11 (18.0)	
N stage				
N0	8	7 (6.3)	1 (1.6)	0.031
N1	36	28 (25.2)	8 (13.1)	
N2	65	43 (38.7)	22 (36.1)	
N3	63	33 (29.7)	30 (49.2)	
Clinical stage				
III	79	55 (49.5)	24 (39.3)	0.206
IVA	93	56 (50.5)	37 (60.7)	
Pretreatment EBV DNA (IU/mL)				
<430	68	55 (49.5)	13 (21.3)	<0.001
≥430	104	56 (50.5)	48 (78.7)	
IC regimens				
TP	97	52 (46.8)	45 (73.8)	0.002
TPF	35	29 (26.1)	6 (9.8)	
GP	40	30 (27.0)	10 (16.4)	

Regarding IC regimens, 97 (56.4%), 35 (20.3%), and 40 (23.3%) patients received TP, TPF, and GP, respectively. In those who received the TP regimen, most patients (*n* = 95, 97.9%) were treated with docetaxel + cisplatin, and only two patients were treated with docetaxel + nedaplatin (2.1%). In those treated with TPF and GP, all patients received cisplatin‐based IC regimens. Moreover, there were 153 (89.0%), 14 (8.1%), and 5 (2.9%) patients were treated with cisplatin‐, lobaplatin‐, and nedaplatin‐based CCRT.

The median plasma EBV DNA was 765 IU/mL (range, 8–90,500 IU/mL), and there were 35.5% (*n* = 61) of patients had plasma residual post_IC_‐EBV DNA. Patients with higher pre_IC_‐EBV DNA were significantly related to a higher rate of plasma residual post_IC_‐EBV DNA (*p* < 0.001). Moreover, patients with the N3 stage had a significantly higher rate of plasma residual post_IC_‐EBV DNA compared to those with N0‐N2 diseases (47.6% vs. 28.4%, *p* = 0.031). Age, gender, smoking history, history, T stage, clinical stage, and IC regimens were not associated with a higher rate of detectable plasma post_IC_‐EBV DNA (Table 1).

The median follow‐up was 30 months (range, 9–55 months). A total of 27 patients had disease recurrence and 19 (70.4%) of them had detectable post_IC_‐EBV DNA. The 3‐year LRFS, DMFS, DFS, and OS in the entire cohort were 92.8%, 87.9%, 82.3%, and 93.7%, respectively.

### 3.2 Association between plasma EBV DNA and survival outcomes

**FIGURE 1:**
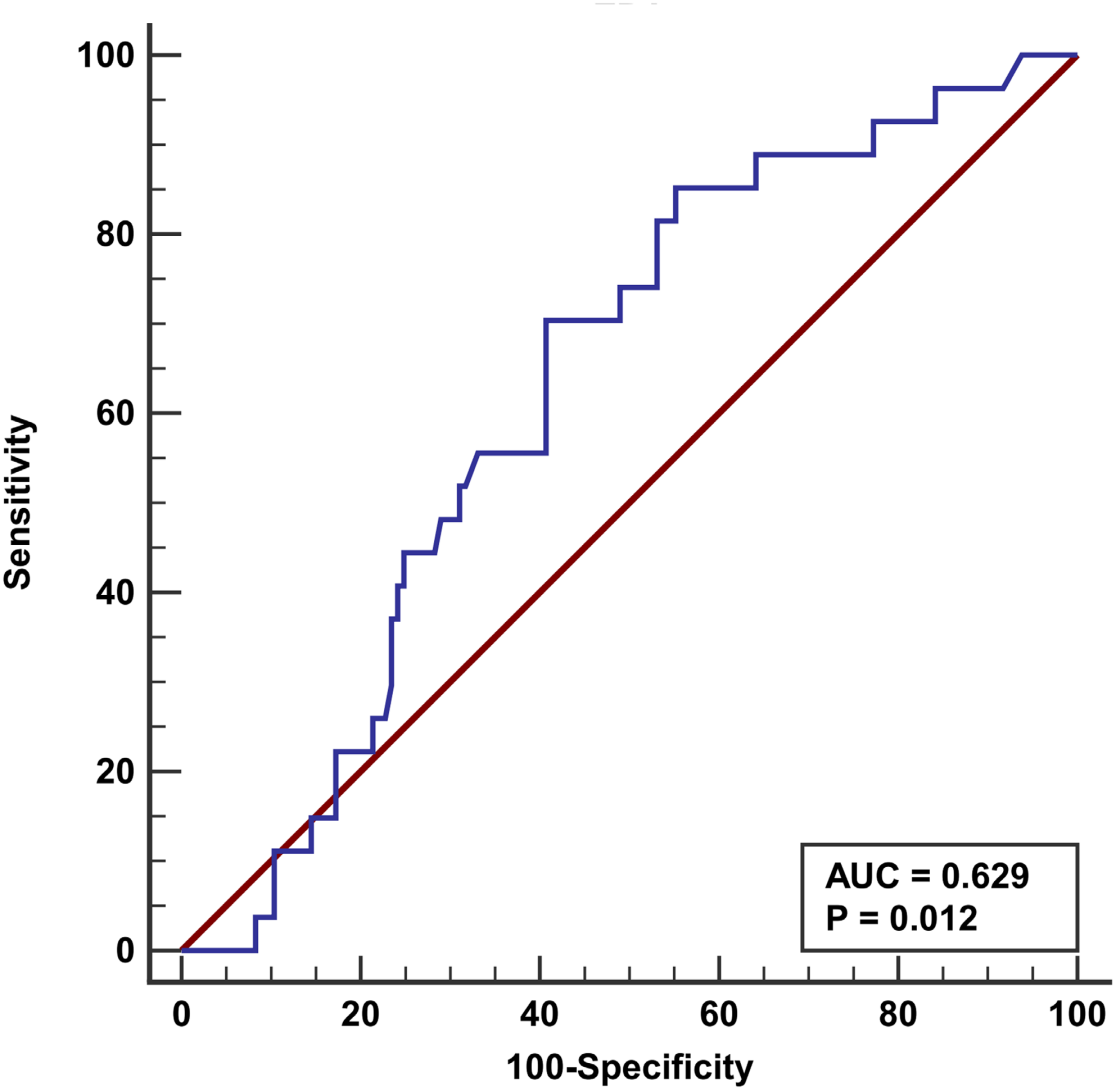
Receiver operating characteristic curve analysis for assessing the optimal cutoff value of pretreatment EBV DNA on 3‐year disease‐free survival.

**FIGURE 2:**
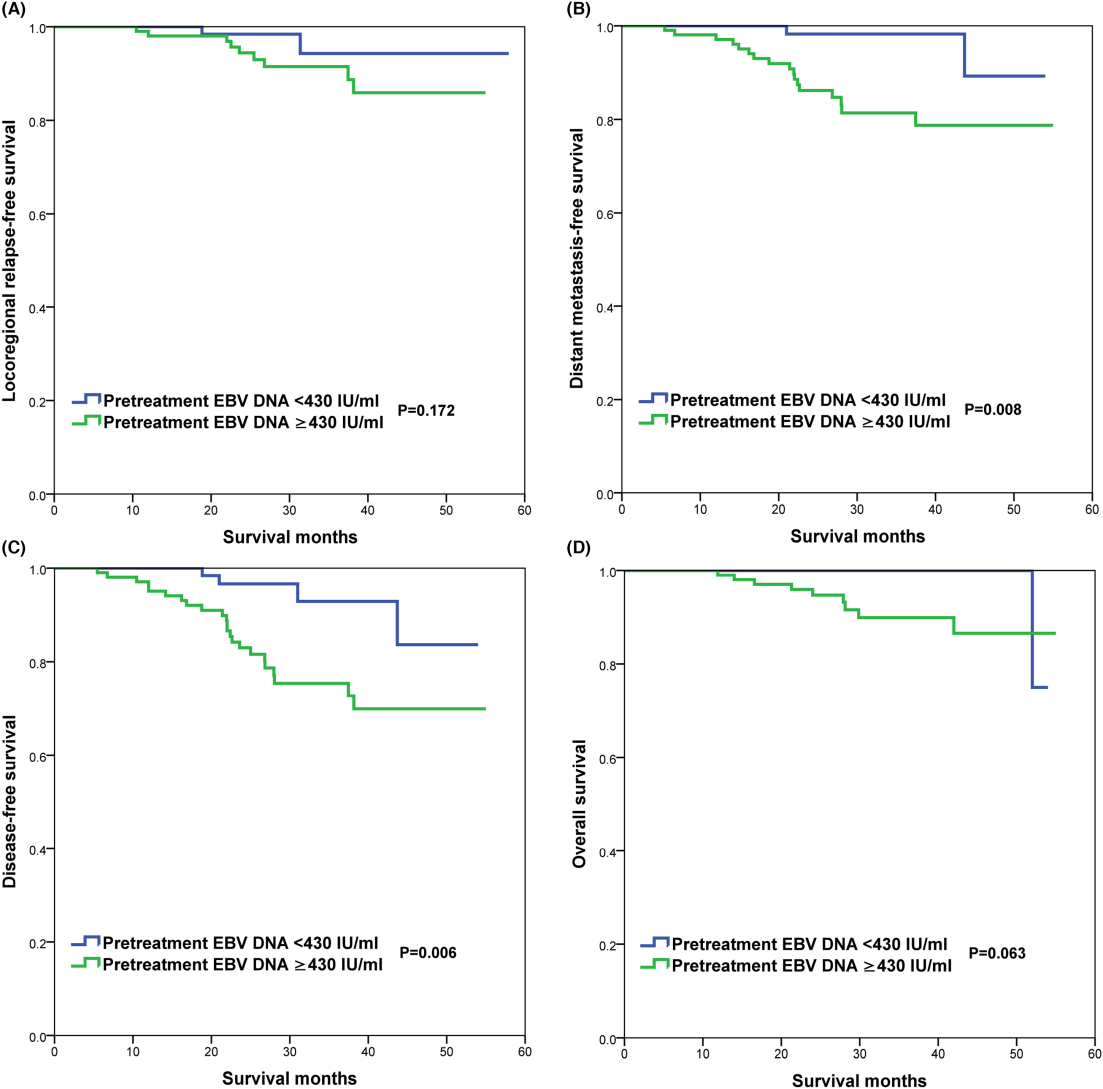
The pretreatment of EBV DNA load in patients with and without residual EBV DNA after induction chemotherapy.

The cutoff value of pre_IC_‐EBV DNA was 430 IU/mL (area under the curve [AUC], 0.629; 95% confidence interval [CI] of the AUC, 0.552–0.701; *p* = 0.012) on 3‐year DFS using the ROC curve analysis (Figure 1). The Kaplan–Meier analysis showed similar LRFS between those with pre_IC_‐EBV DNA <430 IU/mL and ≥430 IU/mL (93.8% vs. 92.4%, *p* = 0.337) (Figure 2A). However, compared with those who had pre_IC_‐EBV DNA <430 IU/mL, those with pre_IC_‐EBV DNA ≥430 IU/mL had inferior 3‐year DMFS (81.4% vs. 9820%, p = 0.008) (Figure 2B) and DFS (75.4% vs. 92.9% *p* = 0.006) (Figure 2C). Regarding OS, those with pre_IC_‐EBV DNA ≥430 IU/mL had comparable OS compared to those with pre_IC_‐EBV DNA <430 IU/mL (89.9% vs. 100%, *p* = 0.063) (Figure 2D).

**FIGURE 3:**
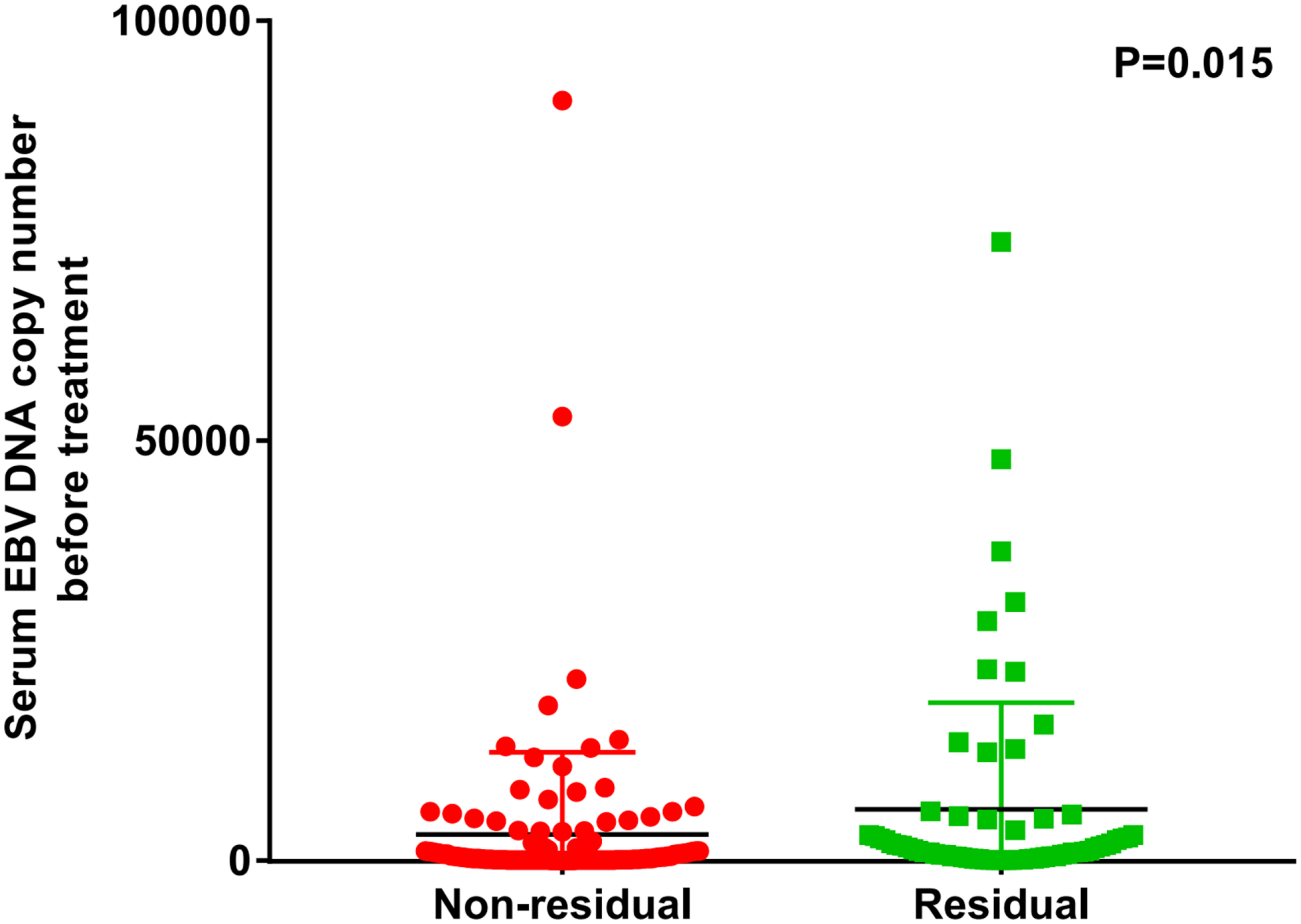
Comparison of survival outcomes between those with pretreatment EBV DNA <430 and ≥ 430 IU/mL (A, LRFS; B, DMFS; C, DFS; D, OS).

**FIGURE 4:**
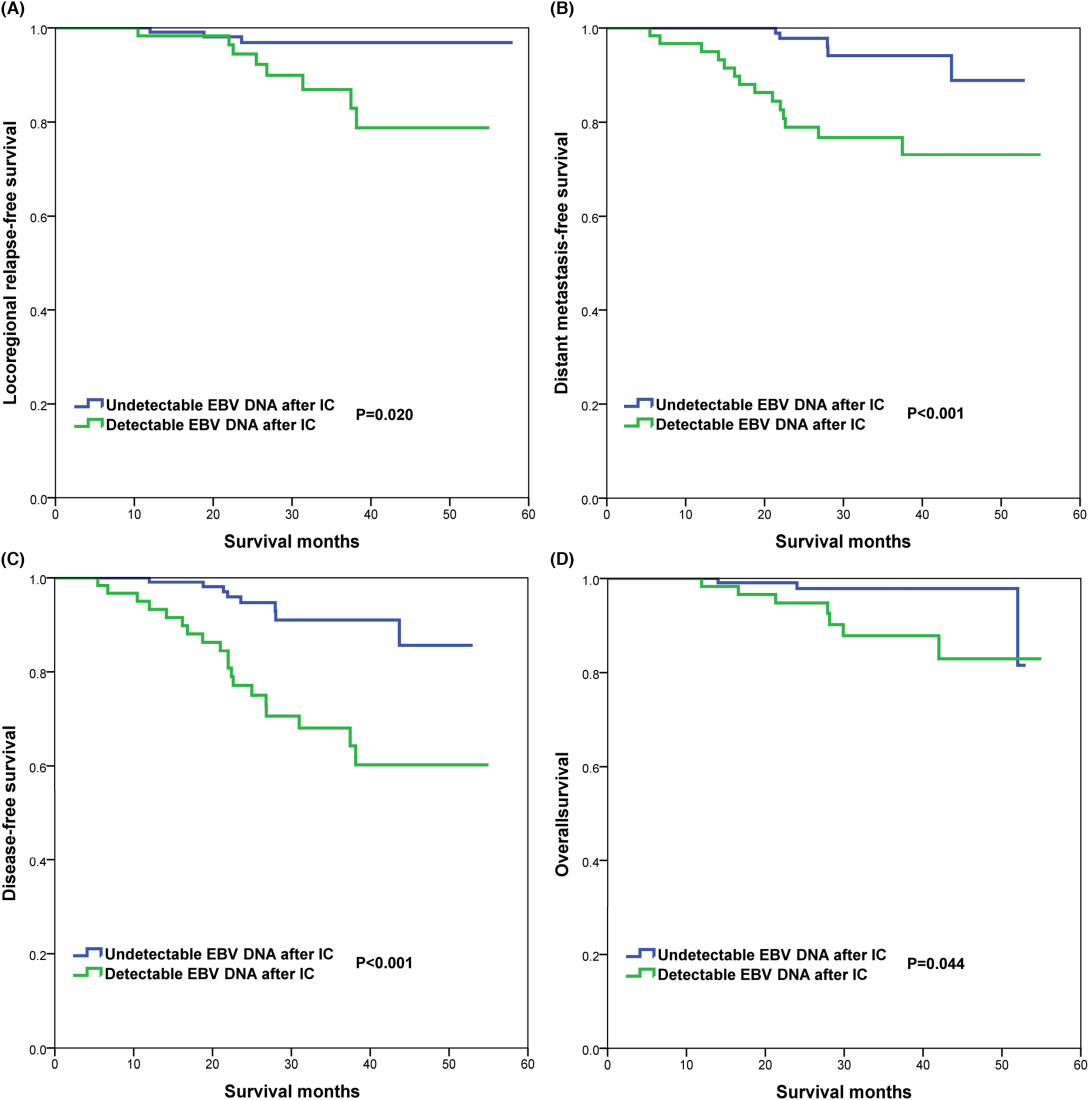
Comparison of survival outcomes between those with and without residual EBV DNA after induction chemotherapy (A, LRFS; B, DMFS; C, DFS; D, OS).

The mean pre_IC_‐EBV DNA in those with and without detectable plasma post_IC_‐EBV DNA was 6498 and 3396 IU/mL, respectively (*p* = 0.015) (Figure 3). Those with detectable post_IC_‐EBV DNA had inferior 3‐year LRFS (86.7% vs. 96.9%, *p* = 0.020) (Figure 4A), DMFS (76.8% vs. 94.2%, *p <* 0.001) (Figure 4B), DFS (68.2% vs. 91.1%, *p <* 0.001) (Figure 4C), and OS (87.8% vs. 97.9%, *p* = 0.044) (Figure 4D) than those with undetectable post_IC_‐EBV DNA. The median time to distant metastasis in patients with detectable post_IC_‐EBV DNA (17.8 months [range, 5–37 months]) was significantly shorter than those with undetectable post_IC_‐EBV DNA (28.0 months [range, 21–44 months]).

### 3.3 Prognostic analyses

The univariate Cox regression analysis showed that detectable post_IC_‐EBV DNA was the only prognostic factor related to LRFS compared to those with undetectable post_IC_‐EBV DNA (hazard ratio [HR] 4.274, 95% CI 1.131–16.148, *p* = 0.032). pre_IC_‐EBV DNA and post_IC_‐EBV DNA were the prognostic factors associated with DMFS and DFS. Age, gender, smoking history, histology, clinical stage, and IC regimens were not associated with survival outcomes (Table S1). The multivariate prognostic analyses showed that detectable post_IC_‐EBV DNA was independently prognostic for lower rates of LRFS (*p* = 0.032), DMFS (*p* = 0.010), and DFS (*p* = 0.004), while it had no significant association with OS (*p* = 0.073) compared to those with undetectable post_IC_‐EBV DNA. The clinical stage was also an independent factor associated with DMFS (HR 2.340, 95% CI 1.835–6.563, *p* = 0.039) (Table 2).

**TABLE 2:** Multivariate analysis in 172 patients with NPC.

Variables	LRFS		DMFS		DFS		OS	
	HR (95% CI)	*p*	HR (95% CI)	*p*	HR (95% CI)	*p*	HR (95% CI)	*p*
Clinical stage								
III	—		1		1		1	
IVA	—	—	2.340 (1.835–6.563)	0.039	2.025 (0.882–4.653)	0.096	3.718 (0.784–17.623)	0.098
Pretreatment EBV DNA (IU/mL)								
<430	—		1		1			
≥430	—	—	3.513 (0.789–15.647)	0.099	2.455 (0.823–7.323)	0.107	—	
EBV DNA after IC								
Undetectable	1		1		1		1	
Detectable	4.274 (1.131–16.148)	0.032	3.949 (1.394–11.191)	0.010	3.504 (1.499–8.192)	0.004	3.455 (0.889–13.423)	0.073

## 4 DISCUSSION

In the current study, we assessed the effect of residual post_IC_‐EBV DNA on the prognosis of LA‐NPC. We found that 35.5% of patients could still detect plasma post_IC_‐EBV DNA, and patients with detectable post_IC_‐EBV DNA had significantly inferior survival outcomes compared to those with undetectable post_IC_‐EBV DNA.

EBV DNA has a strong association with the occurrence and development of NPC.^10^ Currently, research on EBV DNA mainly focuses on its impact on the prognosis of NPC.^23^ In the next version of AJCC staging, EBV DNA may also be integrated into the TNM staging.^24^ However, the optimal threshold of pretreatment EBV DNA level remains controversial. In this study, the median pre_IC_‐EBV DNA load was 765 IU/mL, and 430 IU/mL was the optimal threshold for prognostic analysis. However, the unit of copies/mL was used in the current literature. The median titer of pre_IC_‐EBV DNA load in patients LA‐NPC was 4035–9035 copies/mL.^19^, ^20^ Moreover, the threshold of pre_IC_‐EBV DNA load for predicting survival is wide, ranging from 1000 to 7000 copies/mL.^19^, ^25^, ^26^, ^27^, ^28^, ^29^, ^30^ Conflict results were found from the same institution. In Sun Yat‐sen University Cancer Center, 1500, 4000, or 7000 copies/mL were used for prognostic analysis.^19^, ^27^, ^28^, ^29^, ^30^ However, the results from Hong Kong showed that pre_IC_‐EBV DNA at 500 copies/mL was an optimal threshold for prognostication of NPC.^24^ Therefore, the optimal cutoff values of the pre_IC_‐EBV DNA load are various in different studies. It is difficult to solve this problem unless the standardization between different labs for reporting of plasma EBV DNA load.

As the main treatment strategy of LA‐NPC, IC has been proven to improve the recurrence‐free survival and OS of NPC.^4^, ^5^, ^6^ In clinical practice, the main indicators to predict the survival of patients after IC include pre_IC_‐EBV DNA load, T stage, N stage, and the response to IC.^9^, ^31^ In recent years, several studies have investigated the effect of post_IC_‐EBV DNA in LA‐NPC.^20^, ^26^, ^28^ A study by Jiang et al. found that 28.2% of patients had post_IC_‐EBV DNA. However, the cutoff value of detectable and undetectable EBV DNA was 1000 copies/mL.^26^ In our study, we used 0 as the threshold of undetected EBV DNA. We found that 35.5% of patients still had residual post_IC_‐EBV DNA. In the studies from Zong et al. and Liu et al., they found that 29.6%–32.6% of patients had EBV DNA levels >0 copy/mL after IC,^20^, ^28^ which was similar to our results. We also found that stage N3 diseases had a higher rate of detectable plasma post_IC_‐EBV DNA compared to those with N0‐N2 diseases (47.6% vs. 28.4%, *p* = 0.031). Our finding was similar to the results from Liu et al., who found that 29.2% of patients with stage N0‐2 disease still had residual post_IC_‐EBV DNA, which was significantly lower than those with stage N3 disease (43.8%).^28^

Several previous studies have assessed the role of pre_IC_‐EBV DNA load after radiotherapy on prognostic assessment and adjuvant chemotherapy decision‐making.^12^, ^13^, ^14^, ^15^, ^32^ Peng et al. found that those with a regression rate >95.1% regarding post_IC_‐EBV DNA had a favorable 5‐year OS compared to those with a regression rate ≤ 95.1% (*p* < 0.001).^16^ In our study, we found that those with detectable post_IC_‐EBV DNA had independently associated inferior LRFS, DMFS, and DFS, while pre_IC_‐EBV DNA load was not a risk factor for survival outcomes, which was similar to the findings from Huang et al.^19^ Moreover, we did not find that those with detectable post_IC_‐EBV DNA had significantly lower OS compared to those with undetectable post_IC_‐EBV DNA, but the *p* value was near 0.05 (*p* = 0.073). The lack of significant difference in OS between those with detectable and undetectable post_IC_‐EBV DNA may be due to the limited sample size of patients in our study. The findings from Zong et al. showed that pre_IC_‐EBV DNA load and detectable post_IC_‐EBV DNA were both independent prognostic factors affecting the outcomes of LA‐NPC.^20^ Our study also found that the median time to distant metastasis in patients with detectable post_IC_‐EBV DNA was significantly earlier than in those with undetectable post_IC_‐EBV DNA (17.8 months vs. 28.0 months). Patients with EBV DNA residue found distant metastasis as early as 5 months after treatment, while patients without EBV DNA residue found distant metastasis as early as 21 months. Therefore, the post_IC_‐EBV DNA may be a useful biomarker for better risk stratification and early treatment adjustment of higher‐risk patients before CCRT to improve the treatment effect and ultimately improve survival.

In our study, 27 patients had disease recurrence and 19 (70.4%) of them had detectable post_IC_‐EBV DNA. Moreover, our study also found that detectable post_IC_‐EBV DNA has a significant correlation not only with DMFS but also with LRFS. The potential mechanism of the phenomenon may be related to the relative chemotherapy and radiotherapy resistance in those with detectable post_IC_‐EBV DNA. Huang et al. also found that detectable post_IC_‐EBV DNA had a better effect in predicting survival outcomes compared to those who had detectable EBV DNA after radiotherapy.^19^ Taken together, post_IC_‐EBV DNA may serve as a powerful predictor of survival outcomes compared to pre_IC_‐EBV DNA. To our knowledge, there is still no prospective study on the intensive treatment plan for patients with detectable post_IC_‐EBV DNA. A randomized controlled study from Hong Kong found that the addition of adjuvant chemotherapy to radiotherapy could not improve survival outcomes in patients with detectable plasma EBV DNA after radiotherapy.^33^ According to our results, prospective studies are required to investigate the intensive treatment programs after radiotherapy for patients with residual post_IC_‐EBV DNA. Metronomic therapy may be an effective alternative strategy for high‐risk patients.^32^ Moreover, immunotherapy has achieved significant outcomes in recurrent and metastatic NPC, especially in combined immunotherapy with chemotherapy.^34^

Our study has several limitations. First, there was inevitable selection bias in any retrospective study. Second, the plasma EBV DNA measurements are from a single institution, and international standardization for quantification of EBV DNA load is required to compare the results from different institutions. Moreover, 172 patients for retrospective analysis maybe not enough. Finally, the follow‐up time of our study was short. Despite these limitations, our study added to the current literature regarding the role of plasma post_IC_‐EBV DNA in NPC.

## 5 CONCLUSIONS

In conclusion, our study suggests that the monitoring of plasma post_IC_‐EBV DNA has improved prognostication in LA‐NPC. Our findings suggest that post_IC_‐EBV DNA may be a robust indicator to identify the optimal candidate for intensive treatment. The optimal treatment modality for patients with detectable post_IC_‐EBV DNA needs prospective randomized trials to solve in the future.

## AUTHOR CONTRIBUTIONS

**Hua Zheng:** Writing – review and editing (equal). **Ping Zhou:** Data curation (equal); writing – original draft (equal). **Jun Wang:** Conceptualization (equal); data curation (equal); investigation (equal). **Yi‐Feng Yu:** Formal analysis (equal); investigation (equal); writing – review and editing (equal). **Rui Zhou:** Investigation (equal); methodology (equal); validation (equal); visualization (equal). **Qin Lin:** Conceptualization (equal); project administration (equal); resources (equal); software (equal); supervision (equal); writing – review and editing (equal). **San‐Gang Wu:** Conceptualization (equal); data curation (equal); formal analysis (equal); methodology (equal); project administration (equal); supervision (equal); validation (equal); writing – review and editing (equal).

## CONFLICT OF INTEREST STATEMENT

The authors declare no conflict of interest.

## Supporting information


Table S1.

## Data Availability

Data sharing is not applicable to this article as no new data were created or analyzed in this study.
