# Supplementary material for: Prognostic effect of residual plasma Epstein–Barr viral DNA after induction chemotherapy for locoregionally advanced nasopharyngeal carcinoma
Source: Cancer Med. 2023 May 22;12(14):14979–87. doi: 10.1002/cam4.6132 (PMC10417187; doi:10.1002/cam4.6132)
Supplement: Supplementary file 1 — Table S1. [file CAM4-12-14979-s001.doc]

**Supplementary Table 1**. Univariate analysis in 172 patients with NPC.

| Variables | LRFS | | | DMFS | | | DFS | | | OS | | |
| --- | --- | --- | --- | --- | --- | --- | --- | --- | --- | --- | --- | --- |
|  | HR | 95%CI | P | HR | 95%CI | P | HR | 95%CI | P | HR | 95%CI | P |
| Age (years, continuous variable) | 1.032 | 0.975-1.092 | 0.278 | 1.011 | 0.970-1.053 | 0.616 | 1.010 | 0.981-1.051 | 0.380 | 1.036 | 0.976-1.0101 | 0.246 |
| Gender |  |  |  |  |  |  |  |  |  |  |  |  |
| Male | 1 |  |  | 1 |  |  | 1 |  |  | 1 |  |  |
| Female | 2.031 | 0.589-7.008 | 0.262 | 0.563 | 0.164-1.938 | 0.362 | 0.882 | 0.355-2.193 | 0.787 | 0.394 | 0.050-3.132 | 0.378 |
| Smoking history |  |  |  |  |  |  |  |  |  |  |  |  |
| No | 1 |  |  | 1 |  |  | 1 |  |  | 1 |  |  |
| Yes | 1.153 | 0.350-3.794 | 0.815 | 1.134 | 0.459-2.802 | 0.786 | 1.091 | 0.511-2.329 | 0.822 | 2.191 | 0.564-8.505 | 0.257 |
| Histology |  |  |  |  |  |  |  |  |  |  |  |  |
| WHO I | 1 |  |  | 1 |  |  | 1 |  |  | 1 |  |  |
| WHO II | — | — | 0.973 | — | — | 0.959 | — | — | 0.952 | 0.977 |  | 0.999 |
| WHO III | — | — | 0.971 | — | — | 0.958 | — | — | 0.950 | 24.821 |  | 0.861 |
| Clinical stage |  |  |  |  |  |  |  |  |  |  |  |  |
| III | 1 |  |  | 1 |  |  | 1 |  |  | 1 |  |  |
| IVA | 2.368 | 0.628-8.934 | 0.203 | 2.575 | 0.927-7.153 | 0.070 | 2.215 | 0.969-5.064 | 0.059 | 3.999 | 0.836-19.125 | 0.083 |
| Pretreatment EBV DNA (IU/ml) |  |  |  |  |  |  |  |  |  |  |  |  |
| <430 | 1 |  |  | 1 |  |  | 1 |  |  | 1 |  |  |
| ≥430 | 2.785 | 0.601-12.905 | 0.191 | 5.812 | 1.343-25.161 | 0.019 | 3.922 | 1.356-11.343 | 0.012 | 5.654 | 0.716-44.649 | 0.100 |
| EBV DNA after IC |  |  |  |  |  |  |  |  |  |  |  |  |
| Undetectable | 1 |  |  | 1 |  |  | 1 |  |  | 1 |  |  |
| Detectable | 4.274 | 1.131-16.148 | 0.032 | 5.173 | 1.861-14.376 | 0.002 | 4.382 | 1.917-10.017 | <0.001 | 3.663 | 0.944-14.207 | 0.060 |
| IC regimens |  |  |  |  |  |  |  |  |  |  |  |  |
| TP | 1 |  |  | 1 |  |  | 1 |  |  | 1 |  |  |
| TPF | 1.313 | 0.271-6.368 | 0.736 | 0.591 | 0.132-2.649 | 0.492 | 0.962 | 0.320-2.888 | 0.944 | 2.028 | 0.387-10.613 | 0.403 |
| GP | 0.570 | 0.068-4.807 | 0.605 | 0.743 | 0.208-2.657 | 0.648 | 0.774 | 0.257-2.338 | 0.650 | 1.969 | 0.351-11.049 | 0.441 |
